# S-ketamine promotes postoperative recovery of gastrointestinal function and reduces postoperative pain in gynecological abdominal surgery patients: a randomized controlled trial

**DOI:** 10.1186/s12893-023-01973-0

**Published:** 2023-03-30

**Authors:** Tianzhuo Zhang, Zhijie Yue, Ling Yu, Shuo Li, Yining Xie, Jin Wei, Mengge Wu, Honglei Liu, Hongyu Tan

**Affiliations:** Department of Anesthesiology, Key Laboratory of Carcinogenesis and Translational Research, Ministry of Education/Beijing), Peking University Cancer Hospital & Institute, Haidian District, #52 Fucheng Street, Beijing, 100142 China

**Keywords:** S-ketamine, Gastrointestinal motility, Postoperative pain, Gynecologic surgery

## Abstract

**Background:**

This prospective randomized controlled study was designed to evaluate the effect of S-ketamine with sufentanil given intraoperatively and postoperatively on recovery of gastrointestinal (GI) function and postoperative pain in gynecological patients undergoing open abdomen surgery.

**Methods:**

One hundred gynecological patients undergoing open abdomen surgery were randomized into an S-ketamine group (group S) or placebo group (0.9% saline; group C). Anesthesia was maintained with S-ketamine, sevoflurane, and remifentanil-propofol target-controlled infusion in group S and with sevoflurane and remifentanil-propofol target-controlled infusion in group C. All patients were connected to patient-controlled intravenous analgesia (PCIA) pump at the end of the surgery with sufentanil, ketorolac tromethamine, and tropisetron in group C and additional S-ketamine in group S. The primary outcome was the time of first postoperative flatus, and the secondary outcome was postoperative pain score of patients. Postoperative sufentanil consumption within the first postoperative 24 h and adverse events such as nausea and vomiting were recorded.

**Results:**

The time of first postoperative flatus in group S was significantly shorter (mean ± SD, 50.3 ± 13.5 h) than that in group C (mean ± SD, 56.5 ± 14.3 h, *p* = 0.042). The patient’s visual analog scale (VAS) pain score 24 h after surgery at rest was significantly lower in group S than in group C (*p* = 0.032). There were no differences in sufentanil consumption within the first postoperative 24 h, postoperative complications related to PCIA between the two groups.

**Conclusions:**

S-ketamine accelerated postoperative GI recovery and reduced 24 h postoperative pain in patients undergoing open gynecological surgery.

**Trial registration:**

ChiCTR2200055180. Registered on 02/01/2022. It is a secondary analysis of the same trial.

## Introduction

Opioids remain the most commonly prescribed painkillers for treating severe pain [1,2], despite the potential for adverse side effects that include sedation, dependence, respiratory depression, and bowel dysfunction [3]. The GI related side effect is one of the most common side effects in patients receiving opioids for analgesia [4]. Previous studies have shown that neurogenic, sympathetic efferent nerve pathways, inflammatory, surgical stress, and pharmacological factors may lead to postoperative ileus [5–7]. The use of acetylcholinesterase inhibitors during surgery, such as neostigmine and pyridostigmine, is one effective way to improve gut motility [8–10].

In recent years, the use of ketamine as part of a multimodal analgesia approach to treating acute pain with fewer side effects has gained considerable interest [11]. Ketamine is a non-competitive antagonist of the N-methyl-D-aspartate (NMDA) glutamate receptor that can exist as two enantiomers: R-ketamine and S-ketamine. S-ketamine is twice as potent as ketamine to achieve the same level of sedation [12]. Given this, S-ketamine may achieve the desired analgesic effect while reducing the related psychotomimetic side effects. Several small studies have shown that S-ketamine decreases acute postoperative pain [13–15], but it is not clear if it can reduce postoperative opioid consumption.

NMDA receptors have an important role in the intrinsic neuronal control of GI motility [16]. The expression of NMDA receptors increases peripheral inflammatory reactions, and this upregulation occurs in the neurons of the myenteric plexus [17]. These over-excited neurons can lead to GI hypermotility in inflammatory conditions. Kynurenic acid as one NMDA antagonist effects the motility pattern and inflammation in the examined smooth muscle tone of the colon [18], we speculate that the NMDA glutamate receptors antagonist of S-ketamine may also have a relationship with GI, while it has not been studied extensively in clinical trials.

In this study, we hypothesized that S-ketamine could promote the recovery of GI function and reduce postoperative pain in women undergoing open abdomen surgery with sevoflurane and remifentanil-propofol target-controlled infusion.

## Methods

### Participants

The double-blind, prospective randomized controlled trial was conducted in a tertiary hospital in Beijing, China. This study was approved by the Institutional Review Board at Peking University Cancer Hospital’s Ethics Committee (No.2021YJZ109) and written informed consent was obtained from all subjects participating in the trial. The trial was registered prior to patient enrollment at the Chinese Clinical Trial Registry (No. ChiCTR2200055180, Principal investigator: Hongyu Tan, Date of registration: January 02, 2022). This article adheres to applicable Consolidated Standards of Reporting Trials (CONSORT) guidelines.

Eligible patients were recruited at the Peking University Cancer Hospital from January 2022 to July 2022 if they (1) were aged 18–65 years, with body mass index (BMI, in kg/m^2^) between 18 and 30, were diagnosed with gynecological cancers, and underwent elective open abdominal gynecological surgery, (2) were stratified by the American Society of Anesthesiologists (ASA) grade into I-II, (3) were expected to be hospitalized for over 4 days, and (4) had the operation finished before 20:00 of the day.

All eligible patients were randomly assigned to the S-ketamine group (group S) or placebo (0.9% saline) group (group C) in a 1:1 ratio. The randomization was performed by a data manager who was not involved in determining eligibility or assessment of outcomes. The participants were randomized using random number tables. A research nurse who was independent of the recruitment process opened each patient’s envelope containing the random numbers upon completion of the baseline assessment. Patients with the following conditions were excluded from the study: (1) refusal to participate in the study; (2) tumor recurrence; (3) multiple primary malignant tumors; (4) current and chronic use of analgesics, psychotropic medications, hormones, or non-steroidal anti-inflammatories; (5) a history of chronic pain, schizophrenia, epilepsy, dementia; (6) a history of diabetes with uncontrolled hypertension or blood glucose; (7) hepatorenal dysfunction; (8) any contraindications to the anesthetic agents used during surgery; (9) any of the gynecologic disease such as pelvic inflammatory disease, endometriosis ovarian cyst rupture with hemoperitoneum. The enrolled patients’ age, BMI, ASA physical status, preoperative comorbidity, and type of gynecological cancer were recorded.

### Anesthetic and analgesic techniques

Patient monitoring in the operating room was performed using electrocardiography, pulse oximetry, end-tidal carbon dioxide, invasive arterial pressure, urine output, and the bispectral index (BIS). All patients received general anesthesia induced by intravenous administration of 2 mg/kg of propofol, 0.4 μg/kg of sufentanil, and a single dose of 0.2 mg/kg cisatracurium to facilitate endotracheal intubation. In group C, general anesthesia was maintained by inhalation of 1% sevoflurane and intravenous administration of 2–4 ng/ml (plasma concentration) remifentanil and 1–3 μg/ml (plasma concentration) propofol. In group S, general anesthesia was maintained by the same doses of sevoflurane, remifentanil, and propofol, and an additional target-controlled infusion (CP-600TCI; SLGO, Beijing, China) of 0.2 mg/kg/h S-ketamine (Hengrui Medicine Co., Ltd., Jiangsu, China). The oxygen inhalation flow rate was 50% during anesthesia, and the ventilator was adjusted to maintain the end-tidal carbon dioxide at 35 to 45 mmHg. Sevoflurane and propofol concentrations were adjusted to maintain an adequate depth of anesthesia according to theBIS (spectral entropy value of 40 to 60). Remifentanil concentrations were adjusted by hemodynamic changes. Intravenous sufentanil (10 μg) and ketorolac tromethamine (15 mg) were administered 30 min before the end of the surgery. Tropisetron (5 mg) was administered 15 min before the end of the surgery.

S-ketamine was discontinued 30 min before the end of the surgery. Following surgery, patients were connected to PCIA pump containing 120 ml of 2 μg/kg of sufentanil, 90 mg ketorolac tromethamine, and 20 mg tropisetron in 0.9% sodium chloride solution in group C. An additional 50 mg S-ketamine was added in group S. The PCIA pump was programmed to deliver a loading dose of 2 ml, a 1 ml/h basal infusion, a 2 ml bolus dose with a lockout interval of 10 min, and a maximum dose of 13 ml/h. At the end of the operation, the patient was transferred to the post-anesthesia care unit and then transferred to the ward after extubation. The dose of S-ketamine infusion during surgery, total intraoperative infusion, red blood cell transfusion requirement, blood loss, total urine output, and general anesthesia time were recorded.

### Postoperative data collection

Postoperative pain at rest was assessed using the VAS (ranging from 0 to 10, 0 = no pain, 10 = the worst imaginable pain). Patients received 0.1 μg/kg of sufentanil administered by an anesthesiologist to achieve a VAS score ≤ 3 before discharge from the post-anesthesia care unit. If the VAS score > 3, 0.1 μg/kg sufentanil was given repeatedly in intervals of at least 15 min until the VAS score ≤ 3. Postoperative visits were performed by a nurse anesthetist who was independent of the research process. Additional analgesic drugs (primarily intravenous morphine) were given by surgeon whenever the VAS score was > 3 after pressing the PCIA pump frequently. The S-ketamine consumption of PCIA, first postoperative flatus time of patients, VAS scores at 24 h and 48 h after surgery, the additional requirement of analgesic drugs, and sufentanil consumption of PCIA in the first postoperative 24 h were recorded from the time when the patient was discharged from the post-anesthesia care unit. All adverse events such as dizziness, nausea, and vomiting were documented.

### Sample size

In a preliminary trial that we conducted, the mean of first postoperative flatus time in patients who accepted additional use of S-ketamine was 2.01 days, the standard deviation (SD) was 0.736. The mean of first postoperative flatus time in patients without S-ketamine was 2.54 days, the SD was 0.694. According to PASS 15 power analysis and sample size software (NCSS, LCC. Kaysville, Utah, USA, ncss.com/software/pass.) using a test for two sample T-tests allowing unequal variance with a randomization ratio of 1:1, sample size was 40 patients per group was calculated to provide 90% power to detect this difference based on a two-tailed significance level of 0.05. Considering a dropout rate of about 20%, we intended to enroll 50 patients in each group.

### Statistical analysis

All statistical analyses were performed using Statistical Package for the Social Sciences (SPSS) version 20.0 software (IBM Corp. Released 2011. IBM SPSS Statistics for Windows, Version 20.0. Armonk, NY: IBM Corp.). Continuous variables with normal distribution are presented as mean ± SD. Non-normal variables are presented as median (interquartile range), and categorical data are presented as percentages (%). Continuous variables were analyzed using the independent-sample *t*-test or the Mann–Whitney U test, with differences expressed as 95% confidence interval (CI). Categorical variables were analyzed using the χ^2^ test. A two-sided *P* value of < 0.05 was the statistical significance level.

## Results

**Fig. 1:**
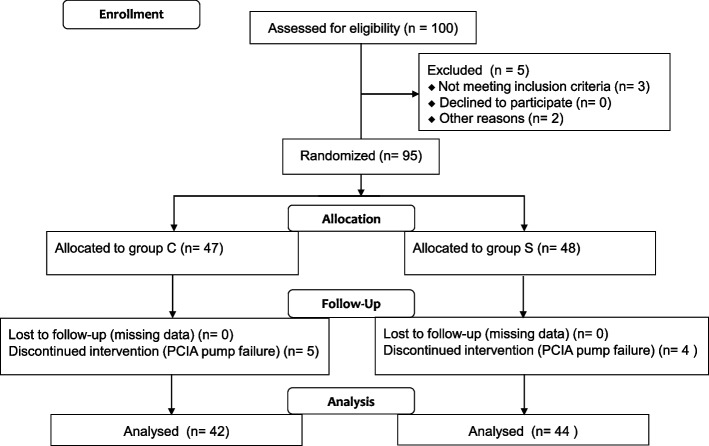
CONSORT flow diagram. CONSORT indicates Consolidated Standards of Reporting Trials. PCIA, patient-controlled intravenous analgesia

A total of 100 patients were recruited for this study from January to July 2022. A total of 14 patients dropped out of the study. Of these, 3 patients were removed due to a change of the operative mode during operation, 2 patients had blood pressure higher than 180/100 mm Hg upon entering the operating room, and 5 patients in group C and 4 patients in group S with PCIA pump failure due to serious side effects within the first 24 h after the operation. Therefore, 86 patients were included in the final analysis. The CONSORT diagram is shown in Fig. 1.

Eighty six patients aged 52.4 ± 6.9 years (range, 33–65) with BMI of 25.2 ± 3.1 (range, 18.9–30.0) had gynecologic cancer and preferred open abdominal surgery were included in this study. There were no statistically significant differences in the demographic data between the two groups (Table 1).

**Table 1:** Patient demographics and perioperative characteristics^a^

	*Group C*^*b*^* (n* = *42)*	*Group S*^*c*^* (n* = *44)*	*95% CI*	*P value*^*d*^
Patient age, mean ± SD, y	52.8 ± 6.4	52.1 ± 7.3	-3.7 to 2.2	0.631^e^
BMI, mean ± SD, kg/m^2^	25.3 ± 3.0	25.1 ± 3.3	-1.5 to 1.2	0.783^e^
ASA physical status, n (%)				
I	14 (33.3)	12 (27.3)	-	0.541
II	28 (66.7)	32 (72.7)		
Preoperative comorbidity, n (%)				
Hypertension	13 (31)	9 (20.5)	-	0.265
Diabetes mellitus	7 (16.7)	3 (6.8)		0.154
Anemia	3 (7.1)	3 (6.8)		0.953
Peripheral neuritis	5 (11.9)	7 (15.9)		0.592
History of surgery	20 (47.6)	24 (54.5)		0.521
Type and FIGO grand of cancer, n (%)				
Cervical cancer	15 (35.7)	9 (20.5)	-	0.148
FIGO grand I	15 (100)	8 (88.9)		
FIGO grand II	0 (0)	1 (11.1)		
Endometrial cancer	8 (19)	6 (13.6)		
FIGO grand I	7 (87.5)	5 (83.3)		
FIGO grand III	1 (12.5)	1 (16.7)		
Ovarian cancer	19 (45.2)	29 (65.9)		
FIGO grand I	1 (5.3)	1 (3.4)		
FIGO grand III	7 (36.8)	15 (51.7)		
FIGO grand IV	11 (57.9)	13 (44.8)		

Abbreviations: *BMI* Body mass index, *ASA* American society of anesthesiologists, *FIGO* International federation of gynecology and obstetrics

^a^Data are presented as mean ± SD or n (%)

^b^Group C: general anesthesia maintained with sevoflurane, remifentanil and propofol, patient-controlled intravenous analgesia pump contained with sufentanil, ketorolac tromethamine, and tropisetron

^c^Group S: general anesthesia maintained with S-ketamine, sevoflurane, remifentanil and propofol, patient-controlled intravenous analgesia pump contained with S-ketamine, sufentanil, ketorolac tromethamine, and tropisetron

^d^Using χ^2^ test, unless indicated

^e^Student *t* test

Intraoperative and anesthetic data are shown in Table 2. The dose of S-ketamine in group S during the operation was 34.1 ± 14.7 mg (range, 13.8–73.2 mg). No statistically significant differences were observed for intraoperative infusion (crystalloids and colloids), red blood transfusion, intraoperative blood loss, intraoperative urine output, or general anesthesia time between groups.

**Table 2:** Intraoperative characteristics^a^

	**Group C**^**b**^** (*n* = 42)**	**Group S**^**c**^** (*n* = 44)**	**95% CI**	***P* value**
Intravascular volume status				
Total intraoperative infusion, M (*P*_25_, *P*_75_), ml	2000 (1500, 2100)	2000 (1600, 2175)	0 to 400 ^d^	0.218^e^
Red blood cell transfusion requirement, n (%)	3 (7.1)	4 (9.1)	-	> 0.999^f^
Blood loss, M (*P*_25_, *P*_75_), ml	200 (100, 300)	200 (100, 400)	0 to 100 ^d^	0.120^e^
Total urine output, M (*P*_25_, *P*_75_), ml	225 (150, 500)	300 (200, 575)	-50 to 100 ^d^	0.406^e^
Duration of general anesthesia, mean ± SD, h	3.6 ± 1.0	3.7 ± 1.1	-0.4 to 0.5	0.879^ g^

^a^Data are presented as mean ± SD, M (*P*_25_, *P*_75_) or n (%)

^b^Group C: general anesthesia maintained with sevoflurane, remifentanil and propofol, patient-controlled intravenous analgesia pump contained with sufentanil, ketorolac tromethamine, and tropisetron

^c^Group S: general anesthesia maintained with S-ketamine, sevoflurane, remifentanil and propofol, patient-controlled intravenous analgesia pump contained with S-ketamine, sufentanil, ketorolac tromethamine, and tropisetron

^d^Hodges–Lehmann estimate based on the Mann–Whitney U test

^e^Wilcoxon rank sum test

^f^χ^2^ test

^g^Student *t* test

The first occurrence of flatus time was significantly different between the two groups (Table 3). Patients in group S had faster flatus time (mean ± SD; 50.3 ± 13.5 h) compared with patients in group C (mean ± SD; 56.5 ± 14.3 h; *P* = 0.042). The patients’ VAS pain score 24 h postoperatively was significantly lower in group S [median 3, interquartile range (IQR), 2 to 4] than in group C (median 3.5, IQR, 2.5 to 4.5, *P* = 0.032). There was no difference in the VAS score 48 h postoperatively (*P* = 0.084).

**Table 3:** Postoperative variables^a^

	**Group C**^**b**^** (*n* = 42)**	**Group S**^**c**^** (*n* = 44)**	**95% CI**	***P* value**
First anal exhaust time, mean ± SD, h	56.5 ± 14.3	50.3 ± 13.5	-12.1 to -2.2	0.042^e^
VAS score after surgery 24 h, M (*P*_25_, *P*_75_)	3.5 (2.5, 4.5)	3 (2.0, 4.0)	-1.0 to 0 ^d^	0.032^f^
VAS score after surgery 48 h, M (*P*_25_, *P*_75_)	2 (1.0, 3.0)	1.5 (1.0, 2.0)	-0.9 to 0 ^d^	0.084^f^
Additional requirement of analgesic drugs, n (%)	2 (4.8)	2 (4.5)	-	> 0.999^ g^
Dose of sufentanil in 24 h postoperative analgesia,				
mean ± SD, μg	56.6 ± 21.1	56.1 ± 19.3	-9.2 to 8.1	0.902^e^

^a^Data are presented as mean ± SD, M (*P*_25_, *P*_75_) or n (%)

^b^Group C: general anesthesia maintained with sevoflurane, remifentanil and propofol, patient-controlled intravenous analgesia pump contained with sufentanil, ketorolac tromethamine, and tropisetron

^c^Group S: general anesthesia maintained with S-ketamine, sevoflurane, remifentanil and propofol, patient-controlled intravenous analgesia pump contained with S-ketamine, sufentanil, ketorolac tromethamine, and tropisetron

^d^Hodges–Lehmann estimate based on the Mann–Whitney U test

^e^Student *t* test

^f^Wilcoxon rank sum test

^g^χ^2^ test

S-ketamine consumption of the PCIA pump in group S in the first postoperative 24 h was 21.8 ± 6.7 mg (range, 8.7–37.1 mg). The additional requirement of analgesic drugs after surgery (*P* > 0.999) and the dose of sufentanil in the first postoperative 24 h (Table 3, *P* = 0.902) were not different between the two groups.

Postoperative complications related to PCIA and the most common side reactions, such as dizziness, nausea, vomiting, and flatulence/bloating were not significantly different between the two groups (Table 4). A total of 40 (46.5%) patients had dizziness, nausea, vomiting, or flatulence/bloating adverse events in this study, 19 (47.5%) of which were in group C, and 21 (52.5%) were in group S. No patient experienced respiratory depression, postoperative delirium, oliguria, or emergence agitation in this study.

**Table 4:** Adverse events and postoperative complications^a^

	**Group C**^**b**^** (*n* = 42)**	**Group S**^**c**^** (*n* = 44)**	***P* value**^**d**^
Adverse events, n (%)	(*n* = 19/42)	(*n* = 21/44)	
Dizziness	11 (26.2)	12 (27.3)	0.910
Nausea	10 (23.8)	11 (25)	0.898
Vomiting	4 (9.5)	9 (20.5)	0.157
Flatulence/Bloating	9 (21.4)	5 (11.4)	0.206
Postoperative complications, n (%)	(*n* = 0/42)	(*n* = 0/44)	
Respiratory depression	0 (0)	0 (0)	-
Postoperative delirium	0 (0)	0 (0)	-
Oliguria	0 (0)	0 (0)	-
Emergence agitation	0 (0)	0 (0)	-

^a^Data are presented as n (%)

^b^Group C: general anesthesia maintained with sevoflurane, remifentanil and propofol, patient-controlled intravenous analgesia pump contained with sufentanil, ketorolac tromethamine, and tropisetron

^c^Group S: general anesthesia maintained with S-ketamine, sevoflurane, remifentanil and propofol, patient-controlled intravenous analgesia pump contained with S-ketamine, sufentanil, ketorolac tromethamine, and tropisetron

^d^χ^2^ test

The basal infusion rate of patients’ PCIA pumps was downregulated to 0.5 ml/h by anesthesiologist when side effects such as dizziness, nausea, vomiting, or flatulence/bloating were serious. Table 5 presents the relevant reasons for the change in PCIA basal infusion rate after surgery. 37 patients required a change in the PCIA pump basal infusion rate due to serious adverse events, 17 (17/42) patients in group C and 20 (20/44) patients in group S. Downregulation of the infusion rate due to dizziness (*P* = 0.912), nausea (*P* = 0.625), vomiting (*P* = 0.375), and flatulence/bloating (*P* = 0.678) were not significantly different between the two groups.

**Table 5:** The reason for the change of PCIA loading dose^a^

	**Group C**^**b**^** (*n* = 17/42)**	**Group S**^**c**^** (*n* = 20/44)**	***P* value**^**d**^
Dizziness, n (%)	9 (21.4)	9 (20.5)	0.912
Nausea, n (%)	6 (14.3)	8 (18.2)	0.625
Vomiting, n (%)	4 (9.5)	7 (15.9)	0.375
Flatulence/Bloating, n (%)	6 (14.3)	4 (9.1)	0.678

Abbreviation: PCIA, patient-controlled intravenous analgesia

^a^Data are presented as n (%)

^b^Group C: general anesthesia maintained with sevoflurane, remifentanil and propofol, patient-controlled intravenous analgesia pump contained with sufentanil, ketorolac tromethamine, and tropisetron

^c^Group S: general anesthesia maintained with S-ketamine, sevoflurane, remifentanil and propofol, patient-controlled intravenous analgesia pump contained with S-ketamine, sufentanil, ketorolac tromethamine, and tropisetron

^d^χ^2^ test

## Discussion

Our preliminary experiment found that the use of S-ketamine shortened patients’ first occurrence of flatus time after surgery by about 0.5 days. This interesting phenomenon has not been previously reported and thus we set the time of first postoperative flatus as the primary outcome in our current study. The randomized controlled trial during open gynecological surgery and the use of a PCIA pump after surgery showed that S-ketamine enhanced recovery of postoperative GI function. The first flatus time in the S-ketamine group was nearly 6 h earlier than in the control group.

The side effects of opioids on the GI tract via the central nervous system and the enteric nervous system have been widely investigated [19]. The δ, κ, and μ opioid receptors have been found in the walls of the stomach, small intestine, large intestine, neurons, and smooth muscle vessels; the μ receptor is the most important in the human enteric nervous system [20]. Opioids inhibit neurotransmitter release, which decreases intestinal propulsive activity [21]. The current study used the opioid anesthetics remifentanil and sufentanil with no significant difference during the operation and PCIA between both groups. Postoperative care was also consistent between the two groups and patients were encouraged to walk slowly on postoperative day 1. Due to the same conditions after surgery in both groups, we speculate that the enhanced recovery time in the S-ketamine group may be due in part to a relationship between NMDA receptors and opioid receptors in enteric neurons. Animal studies have also shown that NMDA receptor antagonists will inhibit opioid release [22], thus preventing many side effects such as paralytic ileus [23].

There are also some studies showed that ketamine on the gastro-intestinal motility was mixed and controversial. One clinical trial of patients undergoing elective surgery of the lower extremities, the use of ketamine-midazolam (250 mg/25 mg; dosage 0.1 ml/kg/h) anesthesia showed no inhibition of intestinal motility compared with fentanyl/midazolam (1 mg/25 mg; dosage 0.1 ml/kg/h) anesthesia [24]. Besides human experiment, animal models about the ketamine effect on intestinal motility was also inconsistent. A study in mice indicated that ketamine could accelerate the intestinal transit in a dose-dependent manner [25], while ketamine provoked no basic changes in gastrointestinal motility in the dog [26], and delayed gastrointestinal transit time in healthy horses [27]. Considered the patients in our trial were undergoing major abdominal surgery and the gastrointestinal motility could be more affected, and the S-ketamine is more effective in analgesic than ketamine, our clinical trial conditions seem to be different with the trial or the experiments mentioned above. Meanwhile, the repeatability and effectiveness of our results still needs to be validated by more studies.

Previous studies mentioned that ketamine with large dose (> 2 mg/kg, iv) and rapid iv administration (> 40 mg/min) can cause hallucinations, unpleasant reactions and nightmares in adults [28–30]. While in our trial, psychotomimetic side effects such as postoperative delirium, oliguria, and emergence agitation were not recorded in patients that accepted S-ketamine during the observation period. These results are consistent with previous studies with sub-anesthetic S-ketamine [31, 32].

Opioids often cause nausea and vomiting, dizziness, and GI disturbance, which are symptoms of chemoreceptor stimulation [33]. One of the most frequent adverse medication reactions linked to opioid use is GI discomfort, which leads to decreased quality of life in patients [4, 34]. Our study showed that the additional application of S-ketamine modestly improved postoperative analgesia and did not increase opioid-related side effects. The incidence of nausea and vomiting in our study was 46.5%. It is noteworthy that 9 patients dropped out of the study for refusal to use the PCIA pump postoperatively due to serious side effects of nausea and vomiting. Thus the actual number of patients that experienced postoperative nausea and vomiting (PONV) was 49 (51.6%) in this study.

Previous reports have shown that females are more sensitive to postoperative pain [35, 36], and gynecological surgeries are associated with a higher risk of PONV [37]. In a randomized, controlled, factorial trial of 5161 patients for the prevention of postoperative nausea and vomiting, 34% of patients had PONV and of these 81.5% were female [38]. Among patients undergoing gynecological surgery in a retrospective observational study, 81 out of 146 patients (55%) developed PONV in 24 h [39]. In another randomized, double-blinded prospective study of 89 patients in open gynecological surgery, nausea and vomiting occurred in 57% [32], similar to our findings here (51.6%). Due to increased adverse effects in females after surgery, more effective anesthetics with fewer side effects should be considered in clinical work.

In our study, all patients received preoperative education and were encouraged to control pain by using PCIA pump. A total of 4 patients received the additional requirement of analgesic drugs, 2 patients in each group. Although, the assumption of sufentanil in the first postoperative 24 h were similar in two groups, our study showed that additional use of S-ketamine reduced the VAS pain score 24 h postoperatively in gynecological abdominal surgery patients. Because of the addition of S-ketamine, the effect of reducing pain becomes reasonable. Previous studies showed that S-ketamine enhanced analgesic efficacy [40, 41].

Our findings indicating no significant difference in sufentanil use in the first postoperative 24 h between the two groups are also consistent with previous studies [32, 42, 43]. In a randomized, double-blinded clinical trial of 30 patients undergoing knee arthroscopy with 0.5 mg/kg bolus of S-ketamine before skin incision and followed by a continuous infusion of 0.12 mg/kg/h until emergence, there was no significant difference in the consumption of morphine in 24 h [43]. In a study of 90 female patients that underwent elective open abdomen hysterectomy with 0.25 mg/kg intravenous boluses of S‑ketamine before skin incision, the cumulative 24 h postoperative morphine consumption was not changed [32].

Contrary to that, several studies have indicated that S-ketamine reduces postoperative opioid consumption [44–46]. In a randomized, double-blind clinical trial of 90 patients undergoing elective cardiac surgery with 75 µg/kg bolus and 0.075 mg/kg/h infusion of S-ketamine, oxycodone consumption during the first postoperative 48 h was reduced in the S-ketamine group [44]. Based on the studies above, it remains unclear if S-ketamine reduces postoperative opioid consumption. In our study, the additional use of S-ketamine did not affect postoperative sufentanil consumption. These inconsistencies may be due to differences in study parameters including the dosage and administration time of S-ketamine, the postoperative VAS scores of patients, and differences in the patient populations of different studies. It is also possible that postoperative opioid consumption is influenced by biological factors as well as social and management factors.

In addition, although we selected open abdomen gynecological surgeries with no intestinal lesions for this study, the gentle touching of the intestine and returning the viscera into the abdominal cavity may have affected the normal physiological morphology of the intestine. Further studies are warranted to confirm the efficacy of S-ketamine to enhance intestinal recovery in patients undergoing intestinal resection surgery.

## Conclusions

In summary, our study provides evidence that an adjunct of S-ketamine shortens the time to the first passage of flatus in patients undergoing gynecological abdominal surgery and is effective for decreasing the intensity of pain within the first 24 h after surgery.

## Data Availability

The datasets used and/or analysed during the current study available from the corresponding author on reasonable request.

## Abbreviations

GI: Gastrointestinal
PCIA: Patient-controlled intravenous analgesia
VAS: Visual analog scale
NMDA: N-methyl-D-aspartate
CONSORT: Consolidated standards of reporting trials
BMI: Body mass index
ASA: American society of anesthesiologists
BIS: Bispectral index
SD: Standard deviation
SPSS: Statistical package for the social sciences
CI: Confidence interval
FIGO: International federation of gynecology and obstetrics
IQR: Interquartile range
PONV: Postoperative nausea and vomiting
